# Kidney Injury Urine Biomarker Normal Ranges in Children

**DOI:** 10.1016/j.ekir.2026.106348

**Published:** 2026-02-04

**Authors:** Hannah Weber, Katharina Schermuly, Anna Tschirner, Ineke Böckmann, Veronika Esslinger, Helene Tietze, Ulrich Baumann, Anibh M. Das, Nele Kanzelmeyer, Jens Drube, Dirk Schnabel, Dieter Haffner, Maren Leifheit-Nestler

**Affiliations:** 1 Department of Pediatric Kidney, Liver, Metabolic and Neurological Diseases, Hannover Medical School, Hannover, Germany; 2 Department of Pediatric Pneumology, Allergy and Neonatology, Hannover Medical School, Hannover, Germany; 3 Center for Chronically Sick Children, Pediatric Endocrinology, University Medicine, Charité Berlin, Berlin, Germany

**Keywords:** CHI3L1, DKK3, EGF, KIM-1, MCP-1, PIIINP

## Abstract

**Introduction:**

Traditional biomarkers, serum creatinine, estimated glomerular filtration rate (eGFR), and albuminuria, used to diagnose kidney disease have limitations in sensitivity and specificity. Urinary biomarkers that are highly sensitive to kidney injury, such as kidney injury molecule-1 (KIM-1), neutrophil gelatinase-associated lipocalin (NGAL), dickkopf-3 (DKK3), chitinase 3-like 1 (CHI3L1), monocyte chemoattractant protein-1 (MCP-1), procollagen type III amino-terminal propeptide (PIIINP), and epidermal growth factor (EGF), have become available for adults. The goal of our study was to develop lambda-mu-sigma (LMS)-based continuous pediatric reference values for these urinary biomarkers.

**Methods:**

A total of 304 children aged 0.1 to 18 years (161 boys) were enrolled in the Hannover reference values for pediatrics (HARP) study. Urinary biomarkers were assessed by enzyme-linked immunosorbent assay (ELISA) and indexed to urinary creatinine levels. LMS-based continuous reference percentiles were generated using the RefCurv software.

**Results:**

LMS-based percentiles were established for urinary KIM-1, NGAL, DKK3, CHI3L1, MCP1, PIIINP, and EGF to creatinine ratios which were all found to be age dependent. The urinary NGAL, DKK3, and MCP-1 to creatinine ratios were also associated with sex. Urinary KIM-1, DKK3, CHI3L1, MCP1, PIIINP, and EGF to creatinine ratios were highest during infancy, followed by a continuous decline until the age of 18 years. The urinary NGAL to creatinine ratio was generally higher in girls and showed 2 peaks, one in infancy and the other at the age of 18.

**Conclusion:**

All urinary biomarkers of kidney health measured were age-, and in part, sex-dependent. The LMS-based continuous pediatric reference percentiles allow calculation of standardized patient z-scores to facilitate test result interpretation in children.

## Graphical abstract

**Figure:**
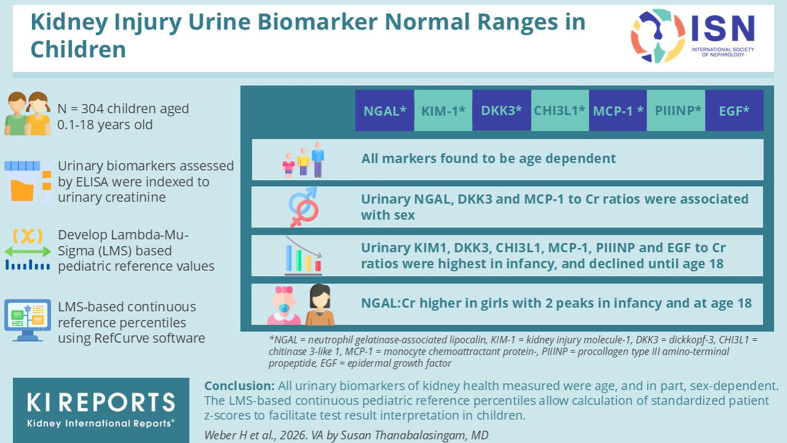

Chronic kidney disease (CKD) is a growing global problem with approximately 700 million people affected worldwide.^1^ Early detection is key in preventing progressive CKD and its associated comorbidity. Traditional biomarkers, such as serum creatinine, calculation of eGFR and albuminuria have limitations in sensitivity and specificity, often failing to detect kidney dysfunction until substantial damage has occurred.^2^^,^^3^ Recently, promising novel biomarkers in urine have been identified, including markers for kidney tubule injury (KIM-1, NGAL, and DKK3), inflammation (CHI3L1, also known as YKL-40, and MCP-1), fibrosis (PIIINP), and tubular health (EGF). They were shown to provide, individually or in combination, earlier and more accurate detection of kidney injury than traditional methods in adults.^4^, ^5^, ^6^, ^7^, ^8^, ^9^, ^10^, ^11^

The use and interpretation of these novel markers in the pediatric population are complicated by the lack of appropriate reference values that account for the physiological maturation of the kidneys. Currently available reference values were measured in small cohorts (< 100 subjects), do not cover the entire pediatric age range, and/or do not take sex-specific differences into account.^12^, ^13^, ^14^, ^15^, ^16^ It is therefore necessary to establish LMS-based continuous reference percentiles for these laboratory parameters, enabling the calculation of patient z-scores and thereby improving data interpretation in clinical practice and studies.^17^, ^18^, ^19^, ^20^ As described by Cole and Green,^17^ the LMS method subjects individual measured values to a smoothing procedure to compensate for random fluctuations in the percentiles and to represent them as a smooth function of age. Thereby, the LMS method assumes that the observed distribution of the measured values (at a fixed age) can be converted into a standard normal distribution using a Box-Cox transformation. The letters L, M, and S stand for skewness (L), median (M), and coefficient of variation (S), where S does not correspond exactly to the coefficient of variation but approximates it. Each of these 3 parameters can vary depending on age, that is, in addition to the mean and dispersion, the distribution assumption can also change over the course of age. The exact age is used as a continuous variable. The 3 parameters L, M, and S are interpreted as a function of (exact) age and modeled as a smooth function using natural cubic splines.

To overcome the current limitations of available kidney injury markers in children, we have generated LMS-based continuous pediatric reference percentiles for 5 novel urinary markers of kidney health as part of the HARP study. These included DKK3, CHI3L1, MCP-1, PIIINP, EGF, and the known markers of tubular kidney damage NGAL and KIM-1. All biomarkers were indexed to urinary creatinine to account for urine concentration.^21^

## Methods

### Participants and Study Design

**Figure 1:**
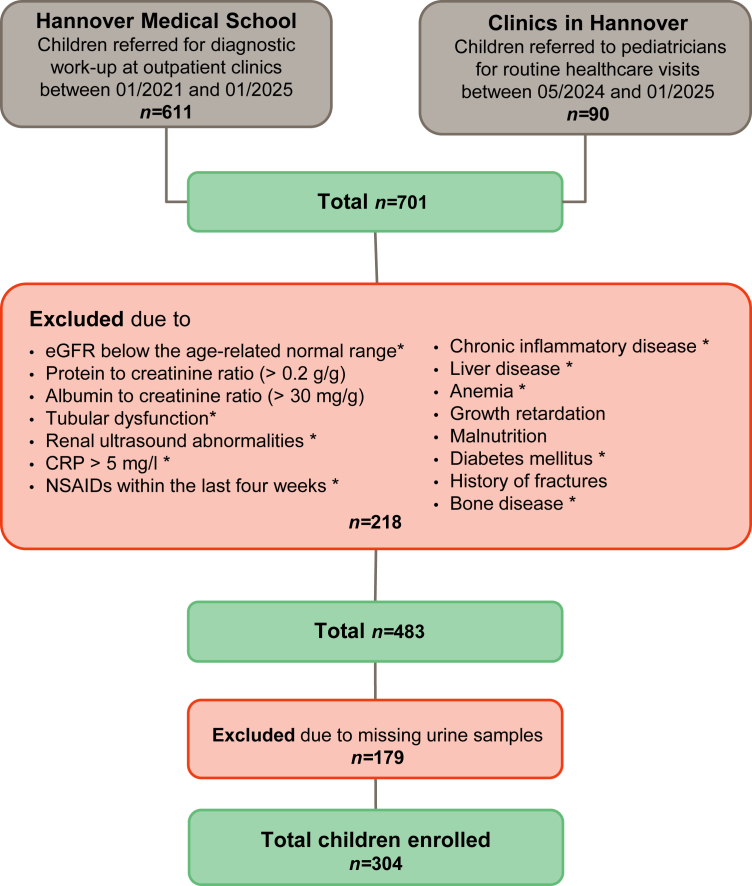
Flow chart showing the selection of participants in the HARP study included urinary biomarker measurements. Exclusion criteria marked with an asterisk (∗) only applies to participants enrolled at Hannover Medical School. CRP, C-reactive protein; eGFR, estimated glomerular filtration; NSAIDs, nonsteroidal anti-inflammatory drugs; HARP, Hannover reference values for pediatrics.

The HARP study was initiated in 2021 to establish LMS-based continuous reference percentiles for key laboratory parameters in children.^18^^,^^19^ This prospective study includes children between the age of 1 month to 18 years, who were either admitted to outpatient clinics at the Children's Hospital of Hannover Medical School in Hannover, Germany, for diagnostic work-up, or healthy children from the Hannover region. Children from the outpatient clinics were defined as healthy and included in the study if they showed no signs of acute illness or urinary tract infections. In the first group, both urine and blood samples were collected to determine serum creatinine, C-reactive protein, alanine aminotransferase, and creatine kinase, whereas in the second group only urine samples were collected. Exclusion criteria were as follows: neonates, growth retardation, malnutrition, bone disease, acute or chronic infection (C-reactive protein > 5 mg/l), inflammatory or liver disease, anemia (hemoglobin levels below the age-related lower limit), evidence or history of acute kidney injury (AKI) or CKD, eGFR below the age-related normal range, abnormalities in kidney ultrasound, proteinuria (protein to creatinine ratio > 0.2 g/g), albuminuria (albumin to creatinine ratio > 30 mg/g), signs of tubular dysfunction (e.g., polyuria and glucosuria), and treatment with nonsteroidal anti-inflammatory drugs or other potentially nephrotoxic drugs (e.g., aminoglycosides) within the last 4 weeks. This analysis is limited to 304 of 701 children and adolescents (161 male) who were assessed for participation in this study by January 2025 and met the above inclusion or exclusion criteria, with at least 10 children (5 boys, 5 girls) per year of life included (Figure 1). Sex was defined as sex assigned at birth and categorized as either male or female. The ethnic background in most participants was Caucasian. The collection of blood and urine samples took place after the first morning urine between 8 am and 2 pm. For infants, urine was collected using urine bags. Serum, plasma, and urine samples were stored at −80 °C until further analysis.

### Anthropometric, Clinical, and Laboratory Measurements

Length (for infants) or height (for older children) and weight were measured using standardized methods to calculate body mass index, and age- and sex-related z-scores for height, weight, and body mass index were calculated using national reference values and the following formula: z = [(x/M)^L^ −1]/S × L.^22^ Automated procedures were used to measure the concentrations of creatinine, cystatin C, C-reactive protein, alanine aminotransferase, and creatine kinase in serum, as well as urinary creatinine, protein, albumin, and glucose. eGFR was calculated using the bedside Schwartz *et al.*’s^23^ formula. Urinary concentrations of KIM-1 (Quantikine ELISA, R&D system; MN Catalog No. DKM100; sensitivity 0.046 ng/ml), NGAL (Quantikine ELISA, R&D system; MN, Catalog No. DLCN20; sensitivity 0.04 ng/ml), DKK3 (Duo Set ELISA, R&D system; MN, Catalog No. DY1118, sensitivity 31.2 pg/ml), CHI3L1 (Quantikine ELISA, R&D system; MN, Catalog No. DC3L10; sensitivity 8.15 pg/ml), MCP-1 (Quantikine ELISA, R&D system;MN, Catalog No. DCP00; sensitivity 10 pg/ml), PIIINP (Novus Biologicals, Wiesbaden Nordenstadt, Germany, Catalog No. NBP2-76434; sensitivity 14.06 pg/ml), and EGF (Quantikine ELISA, R&D system; MN, Catalog No. DEG00; sensitivity 0.7 pg/ml) were measured using ELISA, according to the manufacturer’s protocols. The lowest measurable standard was used for measurements below the detection limit. The samples were measured in duplicate using a Tecan Infinite M200Pro microplate reader (Tecan Life Sciences, Mannedorf, Switzerland) and quantified using Magellan software (version 7.2, Tecan Life Sciences, Mannedorf, Switzerland).

### Statistical Analysis

Data are expressed as median (interquartile range, IQR) or mean ± SD depending on normal distribution, as evaluated by the Shapiro-Wilk test. Comparisons between groups were assessed by the unpaired t test or the Mann-Whitney U test. Concentrations of measured urinary markers were adjusted to urinary creatinine levels and subjected to the LMS method to generate age-specific reference percentiles using RefCurv software (version 0.4.2. for Windows). Data points with 2 SD above or below the 95th and 5th percentile, respectively were identified as outliers and excluded from the construction of the specific percentile curve. The L, M, and S parameters were modeled individually to provide an adequate description of the age-dependent distribution of urinary biomarkers. Model fitting was performed in advanced mode (“Model Fitting – Advanced”). Based on this the 5th, 10th, 25th, 50th, 75th, 90^th^, and 95th percentiles were calculated.^24^ A multivariate linear regression analysis was performed to assess the associations of urine markers with anthropometric data, conventional laboratory parameters, and other urine markers tested. Variables with a significant bivariate correlation (significance level *P* < 0.1) were included in the multivariate linear regression. Data were processed using SPSS (version 29.0.2.0; IBM Corporation, NY).

## Results

### Participants

A total of 304 children (161 boys) with a median age of 9.9 years (IQR 5.9; 13.9) were included in the study (Table 1). Anthropometric parameters adjusted for age and sex, as well as eGFR were within the reference range for healthy children and did not differ between boys and girls, except for the body mass index z-score which was slightly higher in girls compared with boys (0.29 z-score [IQR −0.53; 1.26] vs. −0.06 z-score [IQR −0.68; 0.59], *P* < 0.05).

**Table 1:** Demographic, anthropometric, and biochemical parameters of the HARP cohort

Variables	All (*n* = 304)	Girls (*n* = 143)	Boys (*n* = 161)	*P-*value
Age, yr	9.9 (5.9; 13.9)	11.4 (6.8; 14.8)	9.0 (4.9; 12.4)	0.003
Height, cm	141.0 (116.5; 163.0)	151.5 (120.8; 164.8)	138.0 (110.0; 159.0)	0.068
Height, z-score	0.04 (−0.65; 0.90)	0.10 (−0.53; 0.91)	−0.01 (−0.74; 0.88)	0.391
Body weight, kg	36.7 (20.2; 57.1)	42.6 (22.2; 62.3)	32.2 (18.8; 50.0)	0.007
Body weight, z-score	0.15 ± 1.23	0.28 ± 1.29	0.02 ± 1.17	0.068
BMI, kg/m^2^	17.0 (15.0; 21.0)	19.0 (16.0; 22.0)	17.0 (15.0; 19.0)	< 0.001
BMI, z-score	0.10 (−0.64; 0.78)	0.29 (−0.53; 1.26)	−0.06 (−0.68; 0.59)	0.018
S-crea, μmol/l	49.0 (37.0; 58.3)	50.0 (39.8; 60.0)	44.0 (34.0; 56.0)	0.027
eGFR, ml/min per 1.73 m^2^	110 (98; 123)	108 (98; 122)	114 (98; 124)	0.293

BMI, body mass index; crea, creatinine; eGFR, estimated glomerular filtration rate; HARP, Hannover reference values for pediatrics; IQR, interquartile range; S, serum.

Data are presented as median (IQR) or mean ± SD. *P*-values were calculated using Mann-Whitney U test or unpaired *t* test, respectively.

### LMS Percentiles for Urinary KIM-1, NGAL, DKK3, CHI3L1, MCP-1, PIIINP, and EGF to Creatinine Ratios

**Figure 2:**
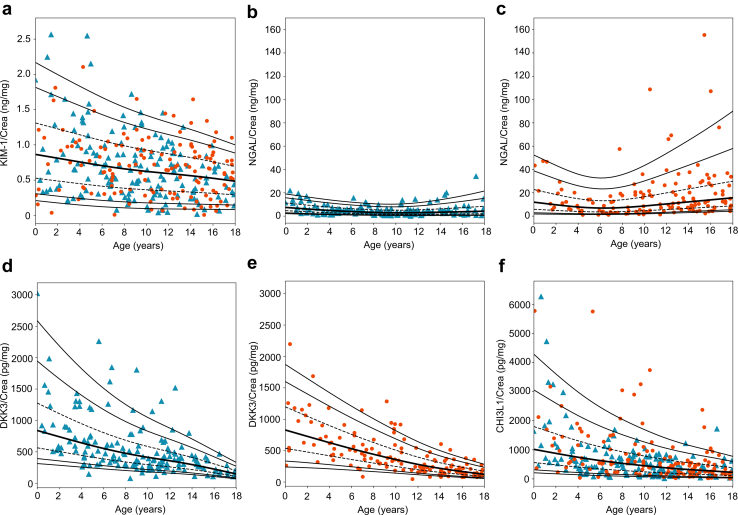
LMS percentiles for urinary (a) KIM-1, (b, boys; c, girls) NGAL, (d, boys; e, girls) DKK3, and (f) CHI3L1 to creatinine ratios in the HARP cohort. The 5th, 10th, 25th (dashed line), 50th (bold line), 75th (dashed line), 90th, and 95th percentiles are given. Data on boys and girls is given as blue triangles and orange dots, respectively. CHI3L1, chitinase 3-like protein-1; crea, creatinine; DKK3, dickkopf-3; KIM-1, kidney-injury molecule-1; LMS, lambda-mu-sigma; NGAL, neutrophil gelatinase-associated lipocalin.

**Figure 3:**
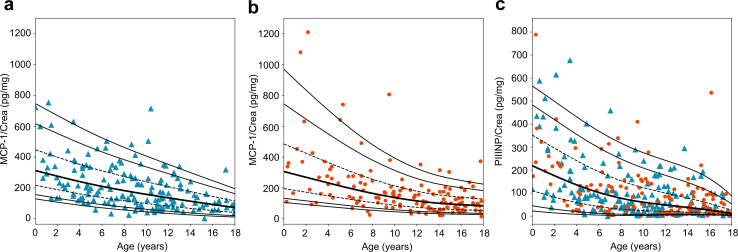
LMS percentiles for urinary MCP-1 (boys, a; girls, b) and (c) PIIINP to creatinine ratios in the HARP cohort. The 5th, 10th, 25th (dashed line), 50th (bold line), 75th (dashed line), 90th, and 95th percentiles are given. Data on boys and girls is given as blue triangles and orange dots, respectively. crea, creatinine; HARP, Hannover reference values for pediatrics; LMS, lambda-mu-sigma; MCP-1, monocyte chemoattractant protein-1; PIIINP, procollagen type III amino-terminal propeptide.

**Figure 4:**
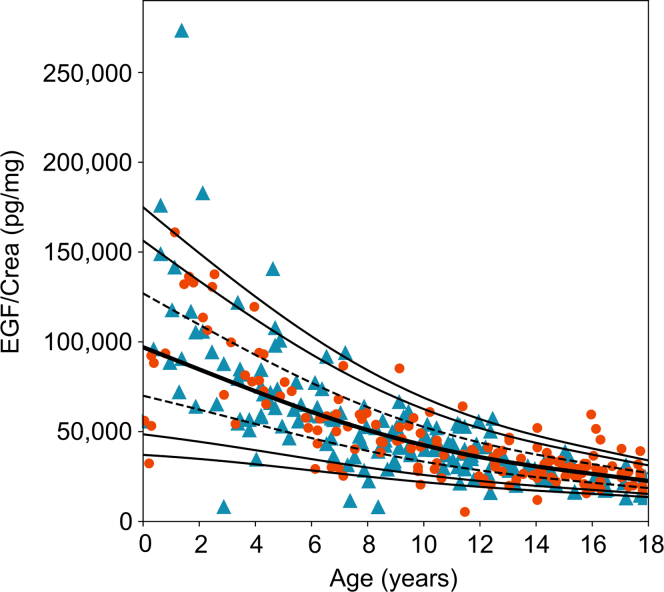
LMS percentiles for EGF to creatinine ratios in the HARP cohort. The 5th, 10th, 25th (dashed line), 50th (bold line), 75th (dashed line), 90th, and 95th percentiles are given. Data on boys and girls is given as blue triangles, and orange dots, respectively. crea, creatinine; EGF, epidermal growth factor; HARP, Hannover reference values for pediatrics; LMS, lambda-mu-sigma.

LMS percentiles were generated for 7 urinary markers of kidney injury, inflammation, fibrosis, and tubule health (Figure 2, Figure 3, Figure 4). All biomarkers investigated were age-dependent (each *P* < 0.05). In addition, the reference values for NGAL (*P* < 0.001), DKK3 (*P* < 0.001), and MCP-1 (*P* = 0.036) were also sex-dependent. Therefore, reference values for the latter parameters are given separately for girls and boys. The respective LMS values are given in Supplementary Tables S1 to S7 in the supplementary material.

The urinary KIM-1 to creatinine ratio reached its peak in infancy, decreased continuously with age, and percentiles converged with increasing age, because of a steeper decline in the 95th percentile compared with the 5th percentile (Figure 2a). The ratio of NGAL to creatinine in urine was generally higher in girls and showed greater variability than in boys. It peaked in the first year of life and then declined steadily until the age of 9 in boys and 6 in girls, followed by a second peak at the age of 18 in both sexes (Figure 2b and c). This age dependence was mainly because of the parabolic shape of the 75th, 90th, and 95th percentiles, which was more pronounced in girls than in boys, whereas the 5th, 10th, and 25th percentiles were unrelated to age.

The urinary DKK3, CHI3L1, MCP-1, PIIINP, and EGF to creatinine ratios showed a similar pattern to the KIM-1 to creatinine ratio (Figures 2d–f, 3a–c, and 4). They exhibited the highest values and variability during infancy, then gradually decreased until the age of 18. In addition, urinary DKK3 and MCP-1 to creatinine ratios differed between boys and girls, with a DKK3 to creatinine ratio higher in boys than in girls, especially in infancy and early childhood (0.1–6 years) (Figure 2d and e; Figure 3a and b). In contrast, the urinary MCP1 to creatinine ratio was higher in girls than in boys, especially in infancy and childhood (0.1–12 years).

### Associations of Urinary Biomarkers With Anthropometric Parameters, and With Each Other

The urinary EGF to creatinine ratio was associated with anthropometric parameters, that is, height, weight, and body mass index z-scores, as well as with urinary KIM-1, DKK3, CHI3L1, MCP-1, and PIIINP to creatinine ratios (cumulative *r*^2^ = 0.543; Table 2). Weaker associations were observed between other urine biomarkers, with an *r*^2^ of 0.068, 0.173, 0.329, 0.336, 0.376, and 0.401 for NGAL, KIM-1, PIIIPN, CHI3L1, MCP-1, and DKK3, respectively.

**Table 2:** Multiple linear regression models of variables associated with urine biomarkers in the HARP cohort

Variable	KIM-1, ng/mg		NGAL, ng/mg		DKK3, pg/mg		CHI3L1, pg/mg		MCP-1, pg/mg		PIIINP, pg/mg		EGF, pg/mg	
	ß	*P*	ß	*P*	ß	*P*	ß	*P*	ß	*P*	ß	*P*	ß	*P*
Height, z-score	--	--	--	--	--	--	--	--	--	--	--	--	−0.683	< 0.001
Body weight, z-score	--	--	--	--	−0.128	0.007	--	--	--	--	--	--	1.061	< 0.001
BMI, z-score	--	--	--	--	--	--	--	--	--	--	--	--	−0.794	< 0.001
KIM-1, ng/mg	--	--	--	--	0.091	0.075	--	--	--	--	--	--	0.289	< 0.001
NGAL, ng/mg	--	--	--	--	--	--	0.198	< 0.001	--	--	--	--	--	--
DKK3, pg/mg	--	--	--	--	--	--	0.170	0.004	0.330	< 0.001	0.227	< 0.001	0.118	0.024
CHI3L1, pg/mg	--	--	0.261	< 0.001	--	--	--	--	--	--	0.205	< 0.001	0.237	< 0.001
MCP-1, pg/mg	--	--	--	--	0.316	< 0.001	--	--	--	--	--	--	0.197	< 0.001
PIIINP, pg/mg	--	--	--	--	0.221	< 0.001	0.205	< 0.001	0.116	0.035	--	--	0.145	0.004
EGF, pg/mg	0.416	< 0.001	--	--	--	--	0.302	< 0.001	0.302	< 0.001	0.285	< 0.001	--	--
*R*^2^ of the regression model		0.173		0.068		0.401		0.336		0.376		0.329		0.543

BMI, body mass index; CHI3L1, chitinase 3-like protein-1; DKK3, dickkopf-3; EGF, epidermal growth factor; HARP, Hannover reference values for pediatrics; KIM-1, kidney injury molecule-1; MCP-1, monocyte chemoattractant protein-1; NGAL, neutrophil gelatinase-associated lipocalin; PIIINP, procollagen type III amino-terminal propeptide.

Values represent regression coefficents (β) and significance levels (p).

## Discussion

This study established LMS-based continuous pediatric reference percentiles for 7 urinary biomarkers for assessing kidney health in the HARP cohort. This included KIM-1, NGAL, DKK3, CHI3L1, MCP-1, PIIINP, and EGF as markers of kidney injury, inflammation, fibrosis, and tubule health, respectively. The LMS percentiles reflect the distinct physiological changes because of kidney maturation and aging in children. The age and/or sex-specific LMS values allow the calculation of reliable standardized patient z-scores for these biomarkers to improve the assessment of kidney health and the early detection of kidney injury.

KIM-1 is mainly expressed in the apical membrane of proximal tubule epithelial cells and highly upregulated in injured kidneys,^4^^,^^25^ ischemic,^25^ and nephrotoxic injury^26^ and accompanied by an increased urinary concentration, compared with healthy subjects. Higher urinary KIM-1 to creatinine values are associated with CKD progression in adults and children with CKD.^11^^,^^27^ Here, we present LMS-based pediatric reference values for urinary KIM-1 to creatinine ratio, which did not differ between the sexes, but were clearly age-dependent. The values decreased progressively with increasing age, mainly because of a sharp decline in the 95th percentile, which was 2.08 ng/mg, 1.42 ng/mg, and 1.00 ng/mg at ages 1, 10, and 18 years, respectively. The presented median of the reference values (0.65 ng/mg creatinine [IQR 0.35; 0.95]) is comparable to those reported in 2 small studies including 25^12^ and 53^13^ Polish children, that is, 0.81 ng/mg creatinine (0.59; 1.01)^12^ and 0.92 ng/mg creatinine (0.44; 1.48),^13^ but substantially lower than the values reported in European adults, with an average age of 62 and 41 years, which were 102 ng/mg creatinine (22; 148)^11^ and 110 ng/mg creatinine (15; 132),^28^ respectively. In all the studies, the same assay was used, which highlights the need for adequate pediatric reference values when assessing urinary biomarkers in children. Our reference values for the urinary KIM-1 to creatinine ratio were higher than those reported in a UK study of 107 Caucasian children (median 0.46 ng/mg creatinine)^14^ and a cohort of 909 Asian children aged between 10 and 18 years (median 0.1 ng/mg creatinine).^15^ The age-related decline in the upper normal values of urinary KIM-1 to creatinine ratio reported in this study was also observed by McWilliam *et al.*^14^ (Meso Scale Discovery, MD) whereas the opposite was found by De Silva *et al.*^15^ (Cusabio Technology, Wuhan, China^15^). This discrepancy could be due to the fact that only children aged between 10 and 18 years were included^15^ in the assay used in the European study.;.^14^^,^^15^

DKK3, a glycoprotein of the DKK1-4 family, is secreted by tubular epithelial cells, particularly under conditions of tubular cell stress or injury. DKK3 modulates the Wnt/β-catenin signaling pathway, which plays an important role in different cellular processes, e.g., proliferation, migration, polarity, and the expression of profibrogenic cytokines.^29^ DKK3 serves as a biomarker for ongoing tubular injury. It is an indicator for short-term CKD progression in adults and children with advanced CKD and for an increased risk for AKI in adults.^16^^,^^29^ Urinary DKK3 is elevated in the early stages of Alport syndrome in children.^30^ The LMS-based reference values for the urinary DKK3 to creatinine ratio in our study were clearly age- and sex-dependent with higher values in boys compared with girls and in younger compared with older children. A previous study of 52 European children (mean age 8.8 ± 3.6 [SD] years) reported a median value for urinary DKK3 to creatinine ratio of 11 pg/mg creatinine.^16^ This is markedly lower than in our reference study (median for 9-year-old boys 454 pg DKK3/mg creatinine and 398 pg DKK3/mg creatinine for girls) and might be due to the different assay used.

The urinary NGAL to creatinine ratio is a well-known marker of kidney injury, as NGAL expression is induced in injured epithelia, and NGAL is excreted into the urine.^31^^,^^32^ Elevated urinary NGAL can be observed in both, AKI and CKD with a tendency to higher levels in AKI than in CKD.^33^ However, LMS-based pediatric reference values were lacking. In this study, the urinary NGAL to creatinine ratios were higher in girls compared with boys, which is in line with 2 European studies by Latoch *et al.*^13^ and McWilliam *et al.*^14^ including 53 and 48 children, respectively. In an Asian study by De Silva *et al.*,^15^ which also used a different assay, no sex differences were noted. The observed age-dependency of urinary NGAL to creatinine reference values in girls was previously noted in European children^14^ but lacking in Asian children.^15^ In contrast to McWilliam *et al.*,^14^ reporting a constant increase in urinary NGAL to creatinine reference values with increasing age, a clear concave age-dependence was noted in this study, which may be due to the use of age categories in the latter study and/or differences in the assay used.

Urinary CHI3L1 and MCP-1 are increasingly used as markers of renal inflammation in adults.^8^ CHI3L1 is a 39 kDa glycoprotein secreted into the urine by activated macrophages under conditions of stress or injury.^34^^,^^35^ Urinary CHI3L1 levels increase in patients after kidney transplantation and AKI.^35^^,^^36^ Nevertheless, CHI3L1 is considered a repair-associated protein, as it inhibits tubular cell apoptosis following AKI.^36^ MCP-1 is a strong chemoattractant for monocytes and is produced by various cell types, including endothelial cells, fibroblasts, mononuclear cells, podocytes, eosinophils, and mast cells. Increased urinary MCP-1 have been reported in various kidney diseases in adults and may serve as a marker for acute renal allograft rejection.^27^^,^^37^ To our knowledge, pediatric reference values for the ratio of CHI3L1 and MCP-1 to creatinine in urine have not been reported previously. However, some studies compared the median values of patient cohorts with those of small groups of healthy children.^38^, ^39^, ^40^, ^41^ The LMS-based reference values given in this study showed a significant decrease with increasing age, with MCP-1 to creatinine ratio also showing higher values in girls compared with boys. Thus, our new data enables the calculation of z-scores for pediatric patients in the future, thereby increasing the sensitivity of these markers for detecting kidney inflammation compared with previous studies.^39^, ^40^, ^41^, ^42^

Collagen type III is synthesized as PIIINP, a 44 kDa protein. Urinary PIIINP to creatinine ratio was associated with renal interstitial fibrosis in adults with various kidney diseases.^42^^,^^43^ Increased urinary PIIINP levels were also associated in children with worsening obstructive nephropathy^44^ and reported in children with solitary kidneys.^45^ Therefore, it may be useful in detecting early and/or progressive kidney impairment in these populations. The LMS-based reference values provided in this study were strongly age- but not sex-dependent, with an about 4-fold higher 95^th^ percentile in infants compared with adolescents, probably reflecting physiological maturation.

EGF plays a crucial role in cell growth, proliferation, and differentiation and is exclusively expressed in distal tubular epithelial cells in the kidneys.^46^ High urinary EGF levels indicate a high regenerative potential and a preserved tubular function.^46^ Conversely, lower urinary EGF levels are associated with more severe tubular atrophy and interstitial fibrosis.^46^ Lower urinary EGF levels were also associated with an increased risk for CKD progression in 2 pediatric cohorts, with mostly nonglomerular types of CKD.^27^^,^^47^ However the latter studies did not include reference values from healthy children. Here we show that urinary EGF to creatinine ratio in healthy children is clearly age-dependent with about 3-fold higher 95th percentiles in infants compared with adolescents aged 14 to 18 years, but does not differ between sexes. An inverse association between urinary EGF to creatinine ratio was previously reported in healthy children and adults with substantially lower reference levels in adults aged above 50 years compared with young adults aged 23 to 30 years. This probably reflects the physiological decline in renal tubular regeneration with increasing age.^21^

Interestingly, significant associations were observed between biomarkers in urine and anthropometric parameters in our cohort of healthy children as well as among urinary biomarker concentrations themselves, which probably reflects the physiological relationship between body growth and kidney maturation and the complex interrelationships between tubular function and regeneration.^48^

Our study has its limitations. Since including healthy children in a reference study like this is difficult, we also included children from outpatient clinics who had to have blood and urine samples taken for diagnostic testing. However, we excluded all children with conditions that could affect kidney health by conducting a thorough medical history review and clinical and laboratory tests. Care must be taken when using our reference values in non-Caucasian children, as only a few non-Caucasian children were included in our study and therefore cannot be generally transferred. For example, the urine KIM-1 reference values for Caucasian children from the UK were significantly higher than those for African-American children from the US.^14^

In conclusion, assessing kidney health in children using urinary biomarkers is challenging, due to marked changes in their urine concentrations during maturation. The provided LMS-based continuous pediatric reference values for important biomarkers of kidney tubule health, injury, inflammation, and fibrosis will allow calculation of standardized patient z-scores to facilitate test result interpretation in children in clinical practice and studies.

## Disclosure

D.H. received speaker fees, consultation fees, and research grants from Kyowa Kirin. I.B. received speaker fees from Kyowa Kirin. All other authors declare no conflict of interest.

## Patient Consent

The study adhered to the principles outlined in the Declaration of Helsinki and received approval from the Ethics Committee of Hannover Medical School (No. 11127). Written informed consent was obtained from all parents or guardians, in addition to age-appropriate consent or assent from the participants themselves.

## Supplementary Material

Supplementary File (PDF) **Table S1.** Age-specific percentile limits and LMS values for KIM-1/crea (ng/mg) independent of sex **Table S2.** Age-specific percentile limits and LMS values for NGAL/crea (ng/mg) in boys and girls. **Table S3.** Age-specific percentile limits and LMS values for DKK3/crea (pg/mg) in boys and girls **Table S4.** Age-specific percentile limits and LMS values for CHI3L1/crea (pg/mg) independent of sex. **Table S5.** Age-specific percentile limits and LMS values for MCP-1/crea (pg/mg) in boys and girls. **Table S6.** Age-specific percentile limits and LMS values for PIIINP/crea (pg/mg) independent of sex. **Table S7.** Age-specific percentile limits and LMS values for EGF/crea (ng/mg) independent of sex.

## Footnotes

Supplementary File (PDF)
**Table S1.** Age-specific percentile limits and LMS values for KIM-1/crea (ng/mg) independent of sex. **Table S2.** Age-specific percentile limits and LMS values for NGAL/crea (ng/mg) in boys and girls. **Table S3.** Age-specific percentile limits and LMS values for DKK3/crea (pg/mg) in boys and girls **Table S4.** Age-specific percentile limits and LMS values for CHI3L1/crea (pg/mg) independent of sex. **Table S5.** Age-specific percentile limits and LMS values for MCP-1/crea (pg/mg) in boys and girls. **Table S6.** Age-specific percentile limits and LMS values for PIIINP/crea (pg/mg) independent of sex. **Table S7.** Age-specific percentile limits and LMS values for EGF/crea (ng/mg) independent of sex.
