# Supplementary material for: Kidney Injury Urine Biomarker Normal Ranges in Children
Source: Kidney Int Rep. 2026 Feb 4;11(4):106348. doi: 10.1016/j.ekir.2026.106348 (PMC12968418; doi:10.1016/j.ekir.2026.106348)
Supplement: Supplementary File (PDF) — Table S1. Age-specific percentile limits and LMS values for KIM-1/crea (ng/mg) independent of sex Table S2. Age-specific percentile limits and LMS values for NGAL/crea (ng/mg) in boys and girls. Table S3. Age-specific percentile limits and LMS values for DKK3/crea (pg/mg) in boys and girls Table S4. Age-specific percentile limits and LMS values for CHI3L1/crea (pg/mg) independent of sex. Table S5. Age-specific percentile limits and LMS values for MCP-1/crea (pg/mg) in boys and girls. Table S6. Age-specific percentile limits and LMS values for PIIINP/crea (pg/mg) independent of sex. Table S7. Age-specific percentile limits and LMS values for EGF/crea (ng/mg) independent of sex. [file mmc1.pdf]

Supplementary material

Kidney injury urine biomarker normal ranges in children

Hannah Weber,[1] Katharina Schermuly,[1] Anna Tschirner,[1] Ineke Böckmann,[1] Veronika Esslinger,[1] Helene Tietze,[1] Ulrich Baumann,[2] Anibh M. Das,[1] Nele Kanzelmeyer,[1] Jens Drube,[1] Dirk Schnabel,[3] Dieter Haffner,[1] and Maren Leifheit-Nestler[1]

[1]Department of Pediatric Kidney, Liver, Metabolic and Neurological Diseases, Pediatric Research Center, Hannover Medical School, Hannover, Germany

[2]Departement of Pediatric Pneumology, Allergy and Neonatology, Hannover Medical School, Hannover, Germany

[3]Center for Chronically Sick Children, Pediatric Endocrinology, University Medicine, Charité Berlin, Berlin, Germany.

Correspondence: Prof. Dr. Dieter Haffner, MD, Department of Pediatric Kidney, Liver, Metabolic and Neurological Diseases, Hannover Medical School, Carl-Neuberg-Str. 1, D-30625 Hannover, Germany. Email: haffner.dieter@mh-hannover.de.

Supplementary Table S1. **Age-specific percentile limits and LMS values for KIM-1/crea (ng/mg) independent of sex**

Age (yrs)	Sex-independent				
	5th	50th (M)	95th	L	S
**0**	0.21	0.86	2.17	0.3758	0.6683
**0.5**	0.20	0.85	2.12	0.3933	0.6700
**1**	0.19	0.84	2.08	0.4109	0.6717
**1.5**	0.18	0.82	2.04	0.4284	0.6733
**2**	0.18	0.81	2.00	0.4460	0.6750
**2.5**	0.17	0.80	1.95	0.4635	0.6767
**3**	0.16	0.78	1.91	0.4811	0.6784
**3.5**	0.15	0.77	1.87	0.4986	0.6801
**4**	0.15	0.76	1.83	0.5162	0.6817
**4.5**	0.14	0.74	1.79	0.5338	0.6834
**5**	0.13	0.73	1.75	0.5513	0.6851
**5.5**	0.13	0.72	1.71	0.5689	0.6868
**6**	0.12	0.70	1.67	0.5864	0.6886
**6.5**	0.12	0.69	1.63	0.6040	0.6903
**7**	0.11	0.68	1.60	0.6215	0.6920
**7.5**	0.11	0.67	1.57	0.6391	0.6937
**8**	0.11	0.66	1.53	0.6566	0.6954
**8.5**	0.10	0.65	1.50	0.6742	0.6972
**9**	0.10	0.64	1.47	0.6917	0.6989
**9.5**	0.10	0.63	1.44	0.7093	0.7006
**10**	0.10	0.62	1.42	0.7268	0.7024
**10.5**	0.09	0.62	1.39	0.7444	0.7041
**11**	0.09	0.61	1.37	0.7619	0.7059
**11.5**	0.09	0.60	1.34	0.7795	0.7076
**12**	0.09	0.59	1.32	0.7970	0.7094
**12.5**	0.09	0.59	1.29	0.8146	0.7111
**13**	0.09	0.58	1.27	0.8322	0.7129
**13.5**	0.09	0.57	1.24	0.8497	0.7147
**14**	0.09	0.56	1.22	0.8673	0.7164
**14.5**	0.09	0.55	1.19	0.8848	0.7182
**15**	0.09	0.54	1.17	0.9024	0.7200
**15.5**	0.09	0.54	1.14	0.9199	0.7218
**16**	0.08	0.53	1.11	0.9375	0.7236
**16.5**	0.08	0.52	1.08	0.9550	0.7254
**17**	0.08	0.51	1.06	0.9726	0.7272
**17.5**	0.08	0.49	1.03	0.9901	0.7290
**18**	0.08	0.48	1.00	1.0077	0.7308

To calculate age-dependent z-scores, the measured concentration x and the age-corresponding values L, M and S must be inserted into the following formula $z = [(x/M)^L - 1]/S \times L$. L, skewness; M, median; S, coefficient of variation; KIM-1, kidney injury molecule-1; crea, creatinine.

Supplementary Table S2. Age-specific percentile limits and LMS values for NGAL/crea (ng/mg) in boys and girls

Age (yrs)	Boys					Girls				
	5th	50th (M)	95th	L	S	5th	50th (M)	95th	L	S
**0**	2.11	7.46	18.99	0.2753	0.6480	1.82	11.96	52.37	0.1456	1.0017
**0.5**	1.98	7.12	18.37	0.2702	0.6571	1.77	11.39	50.16	0.1363	0.9989
**1**	1.86	6.78	17.73	0.2650	0.6663	1.72	10.82	47.94	0.1270	0.9961
**1.5**	1.73	6.44	17.08	0.2599	0.6756	1.67	10.26	45.72	0.1177	0.9932
**2**	1.62	6.10	16.43	0.2547	0.6851	1.62	9.71	43.53	0.1084	0.9904
**2.5**	1.50	5.77	15.77	0.2495	0.6947	1.56	9.18	41.42	0.0991	0.9876
**3**	1.40	5.45	15.13	0.2444	0.7045	1.51	8.69	39.41	0.0897	0.9848
**3.5**	1.30	5.15	14.51	0.2392	0.7144	1.46	8.23	37.57	0.0804	0.9820
**4**	1.20	4.87	13.92	0.2341	0.7244	1.41	7.83	35.96	0.0711	0.9792
**4.5**	1.12	4.60	13.37	0.2289	0.7346	1.38	7.49	34.62	0.0618	0.9764
**5**	1.04	4.36	12.86	0.2237	0.7449	1.36	7.22	33.62	0.0525	0.9736
**5.5**	0.97	4.13	12.39	0.2186	0.7553	1.34	7.04	32.96	0.0432	0.9709
**6**	0.90	3.92	11.96	0.2134	0.7659	1.35	6.93	32.69	0.0338	0.9681
**6.5**	0.84	3.72	11.56	0.2083	0.7767	1.37	6.91	32.82	0.0245	0.9654
**7**	0.79	3.55	11.20	0.2031	0.7876	1.41	6.98	33.38	0.0152	0.9626
**7.5**	0.74	3.38	10.88	0.1979	0.7986	1.46	7.13	34.32	0.0059	0.9599
**8**	0.69	3.24	10.60	0.1928	0.8098	1.53	7.34	35.61	-0.0034	0.9572
**8.5**	0.66	3.12	10.39	0.1876	0.8212	1.61	7.62	37.20	-0.0127	0.9545
**9**	0.62	3.02	10.26	0.1825	0.8327	1.70	7.94	39.06	-0.0221	0.9517
**9.5**	0.60	2.95	10.22	0.1773	0.8444	1.81	8.31	41.16	-0.0314	0.9490
**10**	0.58	2.91	10.28	0.1722	0.8563	1.92	8.70	43.45	-0.0407	0.9463
**10.5**	0.57	2.90	10.44	0.1670	0.8683	2.05	9.12	45.89	-0.0500	0.9437
**11**	0.56	2.91	10.70	0.1618	0.8805	2.17	9.55	48.44	-0.0593	0.9410
**11.5**	0.56	2.95	11.05	0.1567	0.8928	2.31	10.00	51.08	-0.0686	0.9383
**12**	0.55	3.00	11.49	0.1515	0.9054	2.44	10.44	53.80	-0.0779	0.9356
**12.5**	0.56	3.07	12.01	0.1463	0.9181	2.58	10.89	56.57	-0.0873	0.9330
**13**	0.56	3.15	12.62	0.1412	0.9309	2.72	11.33	59.36	-0.0966	0.9303
**13.5**	0.57	3.25	13.29	0.1360	0.9440	2.86	11.77	62.19	-0.1059	0.9277
**14**	0.57	3.35	14.02	0.1309	0.9573	3.01	12.21	65.06	-0.1152	0.9250
**14.5**	0.58	3.45	14.79	0.1257	0.9707	3.15	12.65	67.98	-0.1245	0.9224
**15**	0.59	3.56	15.61	0.1205	0.9843	3.30	13.09	70.95	-0.1338	0.9198
**15.5**	0.59	3.66	16.47	0.1154	0.9981	3.45	13.52	73.99	-0.1432	0.9172
**16**	0.60	3.77	17.38	0.1102	1.0121	3.60	13.96	77.08	-0.1525	0.9146
**16.5**	0.60	3.88	18.34	0.1051	1.0263	3.76	14.39	80.24	-0.1618	0.9120
**17**	0.61	3.98	19.34	0.0999	1.0408	3.91	14.83	83.45	-0.1711	0.9094
**17.5**	0.61	4.09	20.41	0.0947	1.0554	4.07	15.26	86.72	-0.1804	0.9068
**18**	0.62	4.20	21.53	0.0896	1.0702	4.23	15.69	90.07	-0.1897	0.9042

To calculate age-dependent z-scores, the measured concentration x and the age and sex-corresponding values L, M and S must be inserted into the following formula $z = [(x/M)^L - 1]/S \times L$. L, skewness; M, median; S, coefficient of variation; NGAL, neutrophil gelatinase-associated lipocalin; crea, creatinine.

Supplementary Table S3. Age-specific percentile limits and LMS values for DKK3/crea (pg/mg) in boys and girls

Age (yrs)	Boys					Girls				
	5th	50th (M)	95th	L	S	5th	50th (M)	95th	L	S
**0**	297.9	853.6	2499.8	-0.0192	0.6465	239.8	828.4	1872.6	0.4136	0.5898
**0.5**	292.8	828.4	2406.0	-0.0236	0.6401	237.4	803.0	1816.1	0.3995	0.5866
**1**	287.4	803.2	2313.8	-0.0280	0.6338	234.6	777.6	1759.6	0.3854	0.5835
**1.5**	281.9	778.1	2223.2	-0.0323	0.6275	231.4	752.2	1703.1	0.3713	0.5805
**2**	276.2	753.2	2134.4	-0.0367	0.6213	227.8	726.8	1646.6	0.3572	0.5774
**2.5**	270.3	728.4	2047.6	-0.0411	0.6152	223.9	701.5	1590.1	0.3431	0.5744
**3**	264.3	704.0	1962.8	-0.0454	0.6091	219.7	676.3	1533.8	0.3290	0.5713
**3.5**	258.18	679.83	1880.2	-0.0498	0.6031	215.2	651.3	1477.8	0.3149	0.5683
**4**	252.0	656.1	1800.0	-0.0542	0.5971	210.5	626.5	1422.2	0.3008	0.5653
**4.5**	245.8	632.9	1722.3	-0.0586	0.5912	205.5	601.9	1367.2	0.2867	0.5623
**5**	239.6	610.2	1647.4	-0.0629	0.5853	200.3	577.7	1312.8	0.2726	0.5594
**5.5**	233.5	588.1	1575.2	-0.0673	0.5795	194.9	553.8	1259.2	0.2585	0.5564
**6**	227.4	566.7	1505.9	-0.0717	0.5738	189.4	530.3	1206.5	0.2445	0.5535
**6.5**	221.4	546.0	1439.5	-0.0760	0.5681	183.7	507.2	1154.6	0.2304	0.5506
**7**	215.5	526.1	1376.0	-0.0804	0.5625	177.9	484.6	1103.7	0.2163	0.5477
**7.5**	209.8	506.9	1315.4	-0.0848	0.5569	172.1	462.5	1053.8	0.2022	0.5448
**8**	204.2	488.5	1257.9	-0.0892	0.5514	166.2	440.8	1004. 9	0.1881	0.5419
**8.5**	198.9	471.1	1203.5	-0.0935	0.5460	160.1	419.5	956.8	0.1740	0.5391
**9**	193.7	454.5	1152.1	-0.0979	0.5406	154.0	398.5	909.5	0.1599	0.5362
**9.5**	188.8	438.6	1103.4	-0.1023	0.5352	147.8	377.9	862.8	0.1458	0.5334
**10**	184.0	423.5	1057.2	-0.1066	0.5299	141.5	357.5	816.7	0.1317	0.5306
**10.5**	179.4	409.0	1013.1	-0.1110	0.5247	135.1	337.5	771.3	0.1176	0.5278
**11**	174.8	394.8	970.6	-0.1154	0.5195	128.6	317.9	726.9	0.1035	0.5250
**11.5**	170.1	380.7	929.0	-0.1198	0.5143	122.3	298.9	683.9	0.0894	0.5222
**12**	165.2	366.5	887.6	-0.1241	0.5092	116.1	280.8	642.7	0.0753	0.5195
**12.5**	160.0	352.0	845.9	-0.1285	0.5042	110.1	263.6	603.7	0.0613	0.5167
**13**	154.5	336.8	803.5	-0.1329	0.4992	104.4	247.4	566.8	0.0472	0.5140
**13.5**	148.5	321.0	760.1	-0.1372	0.4943	99.0	232.2	532.2	0.0331	0.5113
**14**	142.1	304.5	715.7	-0.1416	0.4894	93.8	217.9	499.7	0.0190	0.5086
**14.5**	135.2	287.3	670.4	-0.1460	0.4845	88.8	204.4	469.	0.0049	0.5059
**15**	127.9	269.5	624.3	-0.1504	0.4797	84.0	191.6	439.91	-0.0092	0.5032
**15.5**	120.2	251.2	577.7	-0.1547	0.4750	79.4	179.4	412.1	-0.0233	0.5006
**16**	112.1	232.5	530.9	-0.1591	0.4703	74.8	167.63	385.1	-0.0374	0.4980
**16.5**	103.8	213.6	484.0	-0.1635	0.4656	70.3	156.2	359.0	-0.0515	0.4953
**17**	95.2	194.4	437.4	-0.1678	0.4610	65.8	144.9	333.3	-0.0656	0.4927
**17.5**	86.4	175.0	391.0	-0.1722	0.4565	61.3	133.9	308.0	-0.0797	0.4901
**18**	77.4	155.6	345.2	-0.1766	0.4520	56.7	122.9	282.8	-0.0938	0.4875

To calculate age-dependent z-scores, the measured concentration x and the age and sex-corresponding values L, M and S must be inserted into the following formula $z = [(x/M)^{L} - 1]/S \times L$. L, skewness; M, median; S, coefficient of variation; DKK3, dickkopf-3; crea, creatinine.

Supplementary Table S4. **Age-specific percentile limits and LMS values for CHI3L1/crea (pg/mg) independent of sex**

Age (yrs)	Sex-independent				
	5th	50th (M)	95th	L	S
**0**	211.43	1016.24	4284.41	0.0580	0.8315
**0.5**	202.29	981.49	4110.40	0.0650	0.8317
**1**	193.28	946.77	3938.95	0.0720	0.8319
**1.5**	184.41	912.12	3770.17	0.0790	0.8321
**2**	175.70	877.64	3604.41	0.0861	0.8323
**2.5**	167.18	843.51	3442.29	0.0931	0.8325
**3**	158.89	809.90	3284.48	0.1001	0.8327
**3.5**	150.88	777.03	3131.71	0.1071	0.8330
**4**	143.16	745.11	2984.70	0.1141	0.8332
**4.5**	135.79	714.33	2844.13	0.1211	0.8334
**5**	128.77	684.85	2710.49	0.1281	0.8336
**5.5**	122.12	656.73	2583.89	0.1351	0.8338
**6**	115.84	630.00	2464.25	0.1421	0.8340
**6.5**	109.90	604.60	2351.30	0.1492	0.8342
**7**	104.28	580.44	2244.47	0.1562	0.8344
**7.5**	98.95	557.34	2142.99	0.1632	0.8346
**8**	93.86	535.12	2046.09	0.1702	0.8348
**8.5**	88.99	513.64	1953.16	0.1772	0.8350
**9**	84.32	492.82	1863.74	0.1842	0.8352
**9.5**	79.83	472.52	1777.33	0.1912	0.8355
**10**	75.50	452.72	1693.77	0.1982	0.8357
**10.5**	71.34	433.42	1613.01	0.2053	0.8359
**11**	67.35	414.73	1535.38	0.2123	0.8361
**11.5**	63.58	396.82	1461.47	0.2193	0.8363
**12**	60.03	379.86	1391.80	0.2263	0.8365
**12.5**	56.71	363.91	1326.59	0.2333	0.8367
**13**	53.63	349.01	1265.86	0.2403	0.8369
**13.5**	50.77	335.15	1209.55	0.2473	0.8371
**14**	48.11	322.21	1157.08	0.2543	0.8373
**14.5**	45.62	310.01	1107.80	0.2614	0.8375
**15**	43.26	298.32	1060.84	0.2684	0.8378
**15.5**	40.98	286.76	1014.82	0.2754	0.8380
**16**	38.72	275.01	968.54	0.2824	0.8382
**16.5**	36.47	262.92	921.57	0.2894	0.8384
**17**	34.22	250.43	873.61	0.2964	0.8386
**17.5**	31.99	237.56	824.82	0.3034	0.8388
**18**	29.79	224.54	775.96	0.3104	0.8390

To calculate age-dependent z-scores, the measured concentration x and the age-corresponding values L, M and S must be inserted into the following formula $z = [(x/M)^L - 1]/S \times L$. L, skewness; M, median; S, coefficient of variation; CHI3L1, chitinase 3-like protein-1; crea, creatinine.

Supplementary Table S5. Age-specific percentile limits and LMS values for MCP-1/crea (pg/mg) in boys and girls

Age (yrs)	Boys					Girls				
	5th	50th (M)	95th	L	S	5th	50th (M)	95th	L	S
**0**	128.86	312.27	745.69	0.0191	0.5336	105.85	305.85	969.86	-0.0759	0.6718
**0.5**	123.30	303.03	726.52	0.0315	0.5390	102.44	296.66	935.40	-0.0697	0.6710
**1**	117.84	293.81	707.22	0.0438	0.5445	99.04	287.46	901.34	-0.0634	0.6702
**1.5**	112.49	284.64	687.86	0.0562	0.5500	95.65	278.25	867.64	-0.0572	0.6694
**2**	107.25	275.56	668.52	0.0686	0.5555	92.27	269.02	834.29	-0.0509	0.6686
**2.5**	102.15	266.60	649.30	0.0809	0.5611	88.90	259.79	801.31	-0.0447	0.6678
**3**	97.21	257.81	630.29	0.0933	0.5668	85.55	250.57	768.75	-0.0384	0.6670
**3.5**	92.42	249.20	611.57	0.1057	0.5725	82.22	241.39	736.67	-0.0322	0.6663
**4**	87.79	240.81	593.22	0.1180	0.5783	78.93	232.27	705.12	-0.0260	0.6655
**4.5**	83.34	232.66	575.29	0.1304	0.5842	75.68	223.22	674.14	-0.0197	0.6647
**5**	79.07	224.78	557.84	0.1428	0.5901	72.47	214.26	643.78	-0.0135	0.6639
**5.5**	74.97	217.15	540.91	0.1551	0.5960	69.31	205.43	614.12	-0.0072	0.6631
**6**	71.04	209.79	524.47	0.1675	0.6021	66.23	196.76	585.26	-0.0010	0.6624
**6.5**	67.28	202.67	508.50	0.1798	0.6082	63.23	188.30	557.33	0.0052	0.6616
**7**	63.66	195.78	492.95	0.1922	0.6143	60.33	180.11	530.46	0.0115	0.6608
**7.5**	60.18	189.09	477.79	0.2046	0.6205	57.54	172.21	504.74	0.0177	0.6600
**8**	56.84	182.59	462.98	0.2169	0.6268	54.87	164.63	480.21	0.0240	0.6592
**8.5**	53.63	176.27	448.48	0.2293	0.6331	52.33	157.39	456.90	0.0302	0.6585
**9**	50.53	170.09	434.25	0.2417	0.6395	49.90	150.47	434.76	0.0364	0.6577
**9.5**	47.55	164.06	420.26	0.2540	0.6460	47.59	143.86	413.73	0.0427	0.6569
**10**	44.67	158.14	406.44	0.2664	0.6525	45.39	137.56	393.77	0.0489	0.6561
**10.5**	41.89	152.31	392.77	0.2788	0.6591	43.30	131.57	374.90	0.0552	0.6554
**11**	39.21	146.58	379.24	0.2911	0.6658	41.33	125.92	357.18	0.0614	0.6546
**11.5**	36.62	140.94	365.81	0.3035	0.6725	39.50	120.64	340.69	0.0677	0.6538
**12**	34.12	135.36	352.46	0.3159	0.6793	37.81	115.78	325.50	0.0739	0.6531
**12.5**	31.70	129.83	339.12	0.3282	0.6862	36.26	111.35	311.66	0.0801	0.6523
**13**	29.37	124.33	325.76	0.3406	0.6931	34.87	107.36	299.20	0.0864	0.6515
**13.5**	27.12	118.85	312.38	0.3529	0.7001	33.63	103.82	288.08	0.0926	0.6508
**14**	24.96	113.42	299.00	0.3653	0.7072	32.53	100.70	278.25	0.0989	0.6500
**14.5**	22.89	108.05	285.70	0.3777	0.7143	31.56	97.96	269.53	0.1051	0.6492
**15**	20.92	102.76	272.51	0.3900	0.7216	30.69	95.52	261.72	0.1113	0.6485
**15.5**	19.06	97.57	259.47	0.4024	0.7289	29.90	93.33	254.64	0.1176	0.6477
**16**	17.31	92.45	246.55	0.4148	0.7362	29.17	91.31	248.11	0.1238	0.6469
**16.5**	15.66	87.40	233.69	0.4271	0.7437	28.48	89.41	241.95	0.1301	0.6462
**17**	14.11	82.37	220.82	0.4395	0.7512	27.82	87.57	236.02	0.1363	0.6454
**17.5**	12.65	77.35	207.84	0.4519	0.7588	27.16	85.74	230.17	0.1426	0.6447
**18**	11.29	72.31	194.73	0.4642	0.7664	26.50	83.91	224.36	0.1488	0.6439

To calculate age-dependent z-scores, the measured concentration x and the age and sex-corresponding values L, M and S must be inserted into the following formula $z = [(x/M)^L - 1]/S \times L$. L, skewness; M, median; S, coefficient of variation; MCP-1, monocyte chemoattractant protein-1; crea, creatinine.

Supplementary Table S6. Age-specific percentile limits and LMS values for PIIINP/crea (pg/mg) independent of sex

Age (yrs)	Sex-independent				
	5th	50th (M)	95th	L	S
**0**	22.36	219.13	565.42	0.9363	1.4389
**0.5**	19.98	208.03	549.11	0.9059	1.4261
**1**	17.76	197.05	532.55	0.8756	1.4133
**1.5**	15.72	186.22	515.79	0.8452	1.4007
**2**	13.84	175.59	498.88	0.8148	1.3882
**2.5**	12.13	165.20	481.91	0.7844	1.3759
**3**	10.59	155.09	464.95	0.7540	1.3636
**3.5**	9.20	145.30	448.11	0.7237	1.3514
**4**	7.97	135.89	431.48	0.6933	1.3394
**4.5**	6.89	126.90	415.21	0.6629	1.3274
**5**	5.95	118.37	399.40	0.6325	1.3156
**5.5**	5.15	110.32	384.12	0.6021	1.3039
**6**	4.47	102.77	369.45	0.5718	1.2922
**6.5**	3.90	95.74	355.41	0.5414	1.2807
**7**	3.45	89.22	342.04	0.5110	1.2693
**7.5**	3.10	83.21	329.35	0.4806	1.2580
**8**	2.85	77.72	317.32	0.4502	1.2467
**8.5**	2.70	72.72	305.98	0.4199	1.2356
**9**	2.66	68.21	295.33	0.3895	1.2246
**9.5**	2.73	64.16	285.36	0.3591	1.2137
**10**	2.90	60.51	276.00	0.3287	1.2028
**10.5**	3.19	57.21	267.14	0.2983	1.1921
**11**	3.54	54.17	258.70	0.2680	1.1815
**11.5**	3.92	51.31	250.56	0.2376	1.1709
**12**	4.28	48.57	242.59	0.2072	1.1605
**12.5**	4.59	45.88	234.65	0.1768	1.1501
**13**	4.83	43.23	226.59	0.1464	1.1399
**13.5**	5.00	40.57	218.26	0.1161	1.1297
**14**	5.10	37.87	209.40	0.0857	1.1196
**14.5**	5.12	35.09	199.70	0.0553	1.1097
**15**	5.06	32.19	188.87	0.0249	1.0998
**15.5**	4.90	29.15	176.66	-0.0055	1.0900
**16**	4.64	25.97	162.83	-0.0358	1.0802
**16.5**	4.28	22.63	147.19	-0.0662	1.0706
**17**	3.82	19.16	129.54	-0.0966	1.0610
**17.5**	3.26	15.58	109.83	-0.1270	1.0516
**18**	2.62	11.95	88.13	-0.1574	1.0422

To calculate age-dependent z-scores, the measured concentration x and the age-corresponding values L, M and S must be inserted into the following formula $z = [(x/M)^L - 1]/S \times L$. L, skewness; M, median; S, coefficient of variation; PIIINP, procollagen type III amino-terminal propeptide; crea, creatinine.

Supplementary Table S7. Age-specific percentile limits and LMS values for EGF/crea (ng/mg) independent of sex

Age (yrs)	Sex-independent				
	5th	50th (M)	95th	L	S
**0**	37.06	97.00	174.95	0.6444	0.4364
**0.5**	36.57	93.92	168.46	0.6399	0.4308
**1**	36.04	90.84	162.03	0.6354	0.4253
**1.5**	35.45	87.75	155.67	0.6309	0.4198
**2**	34.81	84.67	149.38	0.6263	0.4145
**2.5**	34.13	81.60	143.18	0.6218	0.4092
**3**	33.40	78.54	137.06	0.6173	0.4040
**3.5**	32.63	75.51	131.05	0.6128	0.3988
**4**	31.83	72.50	125.16	0.6083	0.3937
**4.5**	31.00	69.54	119.41	0.6037	0.3887
**5**	30.15	66.63	113.81	0.5992	0.3837
**5.5**	29.29	63.79	108.37	0.5947	0.3788
**6**	28.41	61.01	103.12	0.5902	0.3740
**6.5**	27.54	58.33	98.06	0.5856	0.3692
**7**	26.67	55.73	93.22	0.5811	0.3645
**7.5**	25.81	53.24	88.60	0.5766	0.3598
**8**	24.97	50.86	84.20	0.5721	0.3553
**8.5**	24.16	48.59	80.04	0.5675	0.3507
**9**	23.37	46.44	76.10	0.5630	0.3462
**9.5**	22.61	44.40	72.40	0.5585	0.3418
**10**	21.88	42.47	68.91	0.5540	0.3375
**10.5**	21.18	40.65	65.63	0.5494	0.3331
**11**	20.51	38.94	62.56	0.5449	0.3289
**11.5**	19.88	37.33	59.69	0.5404	0.3247
**12**	19.28	35.82	57.00	0.5359	0.3205
**12.5**	18.71	34.41	54.48	0.5314	0.3165
**13**	18.17	33.08	52.13	0.5268	0.3124
**13.5**	17.66	31.84	49.94	0.5223	0.3084
**14**	17.18	30.68	47.88	0.5178	0.3045
**14.5**	16.72	29.57	45.95	0.5133	0.3006
**15**	16.27	28.52	44.11	0.5087	0.2968
**15.5**	15.84	27.51	42.35	0.5042	0.2930
**16**	15.40	26.52	40.63	0.4997	0.2892
**16.5**	14.96	25.53	38.95	0.4952	0.2855
**17**	14.51	24.55	37.28	0.4906	0.2819
**17.5**	14.04	23.57	35.63	0.4861	0.2783
**18**	13.56	22.58	33.98	0.4816	0.2747

To calculate age-dependent z-scores, the measured concentration x and the age-corresponding values L, M and S must be inserted into the following formula $z = [(x/M)^L - 1]/S \times L$. L, skewness; M, median; S, coefficient of variation; EGF, epidermal growth factor; crea, creatinine.
